# ESAT-6 and Ag85A Synthetic Peptides as Candidates for an Immunodiagnostic Test in Children with a Clinical Suspicion of Tuberculosis

**DOI:** 10.1155/2021/6673250

**Published:** 2021-07-06

**Authors:** Zaida Araujo, Carlos Fernández de Larrea, Diana López, Jaime Isern-Kebschull, Jacobus Henri de Waard, Isabel Hagel, Milena Camargo, Magnolia Vanegas, Manuel A. Patarroyo

**Affiliations:** ^1^Laboratorio de Inmunología de Enfermedades Infecciosas, Instituto de Biomedicina “Dr. Jacinto Convit”, Universidad Central de Venezuela, Caracas 1050-58, Venezuela; ^2^Departamento de Hematología, Hospital Clínic, IDIBAPS, Barcelona 08036, Spain; ^3^Servicio de Infectología, Hospital de Niños “J. M. de los Ríos”, Caracas 1050-86, Venezuela; ^4^Departmento de Radiología, Hospital Clínic, Universidad de Barcelona, Barcelona 08036, Spain; ^5^Laboratorio de Tuberculosis, Instituto de Biomedicina “Dr. Jacinto Convit”, Universidad Central de Venezuela, Caracas 1050-58, Venezuela; ^6^Laboratorio de Inmunoquímica, Instituto de Biomedicina “Dr. Jacinto Convit”, Universidad Central de Venezuela, Caracas 1050-58, Venezuela; ^7^Molecular Biology and Immunology Department, Fundación Instituto de Inmunología de Colombia (FIDIC), Bogotá 111321, Colombia; ^8^Animal Science Faculty, Universidad de Ciencias Aplicadas y Ambientales (U.D.C.A), Bogotá 111166, Colombia; ^9^Health Sciences Division, Main Campus, Universidad Santo Tomás, Bogotá 110231, Colombia; ^10^Microbiology Department, Faculty of Medicine, Universidad Nacional de Colombia, Bogotá 111321, Colombia

## Abstract

**Background:**

Tuberculosis (TB) is being underdetected in children as most are smear-negative. This work was aimed at evaluating ESAT-6 and Ag85A synthetic peptides' serodiagnostic potential for diagnosing children having a clinical suspicion of TB.

**Methods:**

The study involved 438 children: 77 Creole nonindigenous (13 suspected of having TB and 64 healthy ones) and 361 Warao indigenous children (39 suspected of TB and 322 healthy children). The approach's diagnostic information was compared using operational characteristics and receiver-operating characteristic (ROC) curves.

**Results:**

Ag85A P-29879 had 94.6% sensitivity (AUC = 0.741: 0.651 to 0.819 95% CI) in indigenous children. ESAT-6 P-12036 and P-12037 had 100% and 92.3% of sensitivity (AUC = 0.929: 0.929: 0.846 to 0.975 95% CI and 0.791: 63.9 to 98.7 95% CI, respectively) in Creole children. ESAT-6 peptides also allowed a differentiation between children with TB and healthy ones.

**Conclusions:**

Further validation of this approach could lead to developing a complementary tool for rapid TB diagnosis in children.

## 1. Introduction

Paediatric tuberculosis (TB) diagnosis research has been very limited even though consensus regarding a reference standard should promote further research [1]; developing new children-related tools has been hampered by a lack of consensus regarding case definitions for research purposes. The World Health Organization (WHO) has estimated that childhood TB accounts for 6% to 10% of all TB cases worldwide every year; more than 74,000 children die from TB every year in countries having a high TB rate and children account for around half a million new cases annually [1].

Child-related multidrug-resistant tuberculosis (MDR-TB) prevention, management, and diagnosis raise special challenges for national TB programmes and are often only accessible at health care referral levels [2]. The lack of an accurate diagnostic TB test for young children is another major challenge and potentially adds to both case underdiagnosis and overdiagnosis [3]. Diagnostic algorithms include scoring systems using clinical parameters and research results; the TB diagnosis “gold standard” consists of a combination of clinical examinations, X-rays, and microscopic and culture-based microbiological tests, the latter possibly taking up to 8 weeks; it should be noted that 10% to 20% of *Mycobacterium tuberculosis* (Mtb) cases are not successfully cultured [4]. Children less than 10 years old have difficulty in producing enough sputum for the standard microscopy test, having low bacterial count; children are thus not considered a major source of infection [5].

Various diagnostic techniques have been developed and evaluated for improving childhood TB diagnosis such as improved culture methods, nucleic acid amplification, and serodiagnosis [6, 7]. Potential Mtb antigen targets were reviewed in a meta-analysis, showing that immunodiagnostic methods using antigen combinations (“cocktails”) had high sensitivity [8, 9]. There has been renewed interest in developing antibody-based diagnostic tests using multiple antigens for achieving high sensitivity and specificity levels [10]. Enzyme-linked immunosorbent assays (ELISA) for TB immunodiagnosis have shown that children have a low humoral response to mycobacterial antigens, especially when combined responses against antigens or specific antibodies (Abs) are not used [11].

An increase of 2.5% was reported regarding TB cases in Venezuela for 2018 (33.2 incidence per 100,000 inhabitants), indigenous people (5.6%) being the most affected. Venezuelan Health Services have reported that the state of Delta Amacuro has had the highest rates since 1999, i.e., 93.2 to 81/100,000 inhabitants. They stated that 90% of these cases have occurred in the Warao indigenous people living in wooden houses raised on stilts along the Orinoco river's banks (Delta's rural areas), adult TB having a very high prevalence [12]. A high TB incidence rate (3,190 per 100,000) has also been reported in Warao indigenous children [13]. A sensitive serological test's positive result giving a reliable active TB diagnosis could be used for diagnosing children having a clinical and epidemiological suspicion of tuberculosis in indigenous and Creole children populations, given the absence of a gold standard test. Such approach would acknowledge that invasive procedures cannot be used for taking samples from indigenous people [14] and that the Warao people have no access to a large hospital in the Delta's rural areas [13, 15].

The TB epidemic in children cannot be ended without advances being made regarding research and development; there is an urgent need for improved diagnostic and treatment options for children suffering TB [3, 5]. This study evaluated the serodiagnosis potential of synthetic peptides covering complete Mtb ESAT-6 and Ag85A antigen sequences, aimed at a rapid diagnosis for children with active TB from two target populations enabling most patients to be identified using a satisfactory cost-benefit ratio.

## 2. Materials and Methods

### 2.1. Study Population

The study involved two populations having different genetic backgrounds; Creole nonindigenous children were prospectively recruited at the “J.M. de Los Ríos” children's hospital in Caracas (Venezuela's capital) and Warao indigenous children recruited from indigenous communities living in a remote rural area in Venezuela's delta region.

The tuberculin skin test (TST) was performed according to international and Venezuelan Tuberculosis Control Programme standards [16]. Children having a ≥10 mm induration were classified as positive [17]. It is worth highlighting that the TST is used as an epidemiological diagnostic test; if positive, it is useful when clinical symptoms suggesting pulmonary TB are also present. The Regional or Delta state TB Control Programme diagnosed children having clinical symptoms suggesting pulmonary TB in indigenous cases. Such symptoms included persistent >38°C fever recorded daily for at least two weeks, persistent cough for more than three weeks, weight loss or failure to thrive, persistent lethargy or decreased playfulness/activity reported by parents, and an absence of clinical response to broad-spectrum antibiotics. The “Dr. Jacinto Convit” Biomedicine Institute's Tuberculosis Laboratory in Caracas diagnosed nonindigenous cases. The children included in this study had met previously reported inclusion criteria [13].

### 2.2. Ethical Approval and Consent to Participate

This study complied with the Helsinki Declaration's principles. It was approved by the Biomedicine Institute-Central University of Venezuela's Research Ethics Committee (protocol number CDCH-UCV-6256-8007/2011). All participating individuals signed voluntary informed consent forms.

### 2.3. Clinical and Laboratory Diagnosis

Sputum samples were collected from indigenous and nonindigenous or Creole children by expectoration; gastric aspirate samples were taken from all Creole children under 6 years of age. Sputum samples were stained for Mtb acid-fast bacilli (Ziehl-Neelsen dye). A sputum smear often does not detect stainable acid-fast bacilli but they can be isolated from sputum by culturing specimens, so confirmed TB was defined as Mtb isolated in culture [18]. The children's standard anteroposterior and lateral chest radiographs (CXRs) were taken for TB confirmation. Indigenous children's X-rays were taken by the “Luis Razzetti” hospital's X-ray service in Tucupita, Delta Amacuro's state capital. Creole children's X-ray studies were made in the “J.M. de Los Ríos” children's hospital in Caracas, Venezuela's capital.

Standard anteroposterior and lateral CXRs were taken for all children; an independent expert evaluated the CXRs. Lacking bacteriological confirmation, a diagnosis of TB was only made when the CXR showed lesions suggestive of active pulmonary TB and a child had a positive clinical and radiographic response to anti-TB treatment. Healthy children without evidence of clinical symptoms suggesting pulmonary TB infection were used as controls. These healthy controls include both the noninfected individuals, as well as children with latent TB (but healthy). HIV was ruled out in child patients and controls by blood tests and active pulmonary TB by CXR. Children should not be taking immunosuppressive drugs due to their effect on the immune response (i.e., corticosteroids, azathioprine, and cyclophosphamide). Children meeting the inclusion criteria were included after their parents had signed an informed consent agreement. TB patients were sampled before beginning anti-TB treatment.

### 2.4. Serodiagnosis

Synthetic peptide ESAT-6 and Ag85A full-length sequences were synthesised at the Fundación Instituto de Inmunología de Colombia (FIDIC) in Bogotá, Colombia, and used as single peptides.  Table 1 shows five ESAT-6-peptides and seventeen Ag85A peptides. The solid-phase multiple peptide system was used for synthesising peptides based on *M. tuberculosis* ESAT-6 and Ag85A amino acid (aa) sequences [19, 20].

Peptide-based indirect ELISA was used for determining IgG reactivity against ESAT-6 and Ag85A peptides in serum, as previously reported [21]. Briefly, sera were obtained from child controls and patients' venous blood. ESAT-6 or Ag85A synthetic peptides at 1 *μ*g/well were then used as antigens to coat 96-well microtitre plates. Indirect ELISA assays were carried out and standardised in our laboratory for measuring Ab (IgG) reactivity against peptides. Standardised serum sample dilution was 1 : 200. After incubating sera for one hour, the plates were washed four times and then incubated with a peroxidase-conjugated goat anti-human IgG Ab (Promega Corporation, US). The plates were then washed four times, and a substrate solution consisting of citrate buffer at pH 5.0, 30% H_2_O_2_, and 10 mg o-phenylenediamine dihydrochloride (OPD, Sigma-Aldrich) was added. The plates were incubated for 6 min at room temperature. An ELISA microplate reader was used for measuring colour development at 492 nm.

### 2.5. Statistical Analysis

Receiver-operating characteristic (ROC) curves were constructed for comparing the methods' overall diagnostic information. Student's *t*-test was used for comparing average age amongst groups. Fisher's exact test was used for comparing significance between percentages for individuals proving positive and/or negative by TST. Considering the distribution, the data was presented in terms of mean or median along with their corresponding dispersion measures (standard deviation—SD or interquartile ranges—IQR). The Mann-Whitney *U* test (nonparametric) was used for comparing differences regarding isotype reactivity amongst groups; raw *p* values were corrected with the Bonferroni method for multiple comparisons [22]. STATA14 software was used for such analysis; significance was set at ≤0.05 *p* value.

## 3. Results

The study involved 438 children aged 1 to 15 years-old; 196 were male and 242 females. The study involved 77 Creole nonindigenous children from Caracas, Venezuela, 16.9% (*n* = 13) being Creole child patients (CPCh) suspected as having TB and 83.1% (*n* = 64) Creole child controls (CCCh). This group was compared to 361 Warao indigenous children from indigenous communities living in a remote rural area in Venezuela's delta region delta region: 10.8% (*n* = 39) Warao child patients suspected of having TB (WPCh) and 89.2% (*n* = 322) Warao child controls (WCCh).

Table 2 lists participants' demographic and clinical data. The mean age for indigenous children having active pulmonary TB was 7.5 (SD = 4.2) and 5.2 (SD = 3.6) for Creole children. Mean control group age was 8.6 (SD = 2.3) for indigenous children and 6.6 (SD = 2.4) for Creole children (Table 2). There were no statistically significant differences regarding gender distribution in the WPCh group (females 41%: 16/39; males 58.9%: 23/39) or the CPCh group (females 46.1%: 6/13 males; 53.8%: 7/13) (Table 2). A statistically significant difference was found between females (195/322: 60.5%) and males (127/322: 39.4%) (*p* < 0.0001) in the WCCh control group compared to the CCCh group (females 25/64: 39%; males 39/64: 60.9%, *p* < 0.02) (Table 2).

Statistically significant differences between the WPCh (74.0%), CPCh (45.5%), (*p* < 0.0001), and WCCh groups (20.1%) (*p* < 0.0001) were found regarding skin test reactivity for studying delayed-type hypersensitivity (Table 2). Ascertaining Creole control children's reactivity to a skin test was not possible because their parents did not agree to them being tested. WPCh group smear sensitivity was 3.7% and culture sensitivity 7.4%, whilst bacteria were not detected in the CPCh group (Table 2). A statistically significant difference was found regarding X-ray characteristic of lesions suggestive of active pulmonary TB between indigenous children (59.3%) and Creole nonindigenous children (100%), *p* < 0.007 (Table 2). Figure S1a (indigenous children) and Figure S1b (nonindigenous or Creole children) give the lesions' radiographic characteristics suggestive of active pulmonary TB.

**Figure 1:**
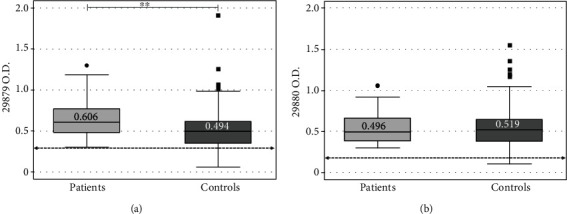
Optical density (OD) value distribution concerning antibodies reactivity between Warao children having active pulmonary TB (*n* = 39) and healthy controls (*n* = 322). (a) IgG reactivity against Ag85A P-29879, -cut-off (>0.381), *p* value < 0.01 (^∗∗^). (b) IgG reactivity against Ag85A P-29880, cut-off (>0.29).

Median IgG reactivity distribution regarding against synthetic peptides was compared; Warao patient children's IgG reactivity against Ag85A P-29879 was significantly higher (0.606 ± 0.289) than that for indigenous controls (0.494 ± 0.257) (*p* < 0.017 Bonferroni correction) (Figure 1(a)). There was no statistically significant difference between indigenous child patients (0.496 ± 0.270) and indigenous controls (0.519 ± 0.269) concerning IgG reactivity against Ag85A P-29880 (*p* = 1.824, Bonferroni correction) (Figure 1(b)).

**Figure 2:**
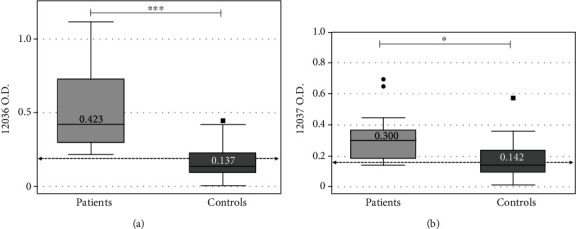
Optical density (OD) value distribution concerning antibodies reactivity comparing Creole children having active pulmonary TB (*n* = 13) to healthy controls (*n* = 64). (a) Peptide 12036 IgG reactivity and anti-ESAT-6, cut-off (>0.204) *p* value < 0.0001 (^∗∗∗^). (b) Peptide 12037 IgG reactivity and anti-ESAT-6 cut-off (>0.146), *p* value < 0.05 (^∗^).

Creole child patients (0.423 ± 0.430) had significantly higher IgG reactivity against ESAT-6 P-12036 than Creole controls (0.137 ± 0.135) (*p* < 0.0001 Bonferroni correction) (Figure 2(a)). There was a statistically significant difference between Creole child patients (0.300 ± 0.179) and Creole controls (0.142 ± 0.102) concerning IgG reactivity against P-12037 (*p* < 0.05 Bonferroni correction) (Figure 2(b)).

The diagnostic accuracy of the peptides included in this study was evaluated; sensitivity for indigenous children ranged from 32.4% (P-11003) to 100% (P-29880); despite the latter's high sensitivity, the AUC was lower: 0.506 ± 0.058; 0.411 to 0.601, 95% CI (data not shown). Its specificity dropped to 20.8%, and it had 1.26 positive likelihood ratio and 0.0 negative likelihood ratio. Positive predictive value was 37.8 and negative predictive value 100 (Table 3).

Ag85A P-29879 also had high sensitivity (94.6%) and specificity (49.4%) (Table 3) and AUC: 07410 ± 0.520.651-0.819 95% CI (Figure S2). Positive likelihood ratio was 1.87 and negative likelihood ratio 0.11; positive predictive value was 47.3 and negative predictive value 95.0 (Table 3). The third peptide Ag85A P-10997 also had high sensitivity (94.6%, similar to P-29879); however, it had a lower AUC (0.532 ± 0.058: 0.436-0.626 95% CI) (data not shown). Its specificity dropped to 16.9%. Positive likelihood ratio was 1.14 and negative likelihood ratio 0.32; positive predictive value was 35.4 and negative predictive value 86.7 (Table 3).

Regarding tests having the good specificity in the indigenous children, Ag85A P-11003 had 97.4% specificity (Table 3) and an AUC 0.573 ± 0.056 477-0.665 95% CI (Figure 2). Positive likelihood ratio was 12.49 and negative likelihood ratio 0.69. Positive predictive value was 85.7 and negative predictive value 75.0 (Table 3). ESAT-6-P-11004 had 93.5% specificity (AUC 0.763 ± 0.044: 0.675-0.838 95% CI). Positive likelihood ratio was 7.91 and negative likelihood ratio 0.52. Positive predictive value was 79.2 and negative predictive value 80.0 (Table 3).

The Creole child group's test sensitivity ranged from 36.4% (P-29881) to 100% (P-12036). Three antipeptide tests revealed the best performance characteristics regarding Creole children having active TB. ESAT-6 P-12036 had the highest sensitivity (100%) and specificity (74.6%) and AUC 0.929 ± 0.050 (Table 3 and Figure S2). Positive likelihood ratio was 3.94 and negative likelihood ratio 0.0. Positive predictive value was 44.8 and negative predictive value 100. ESAT-6 P-12037 also had high sensitivity (92.3%) and specificity (55.6%) (Table 3) and AUC 0.791 ± 0.078: 0.593 to 0.812 95% CI) (data not shown). Positive likelihood ratio was 2.08 and negative likelihood ratio 0.14. Positive predictive value was 30.0 and negative predictive value 97.2 (Table 3). The lower sensitivity (84.6%) and specificity (65.1%) was found to ESAT-6 P-12035. Positive likelihood ratio was 2.42 and negative likelihood ratio 0.24. Positive predictive value was 33.3 and negative predictive value 95.3 (Table 3). Diagnostic performance data for other peptides are shown in Table 3 and Figure S2.

## 4. Discussion

Despite improvements in the global fight against TB, about 30% of ill individuals are underdiagnosed or underreported; such scenario is even worse regarding childhood due to characteristics such as the large percentage of false negatives (consequence of a lower bacillary load), the inability to expectorate sputum, and because several common diseases during childhood can mimic TB clinical presentation. Paediatric clinical isolates contain fewer bacteria (paucibacillary), making culture and isolation even more challenging. Whilst bacterial culture is considered gold standard of TB diagnostic in adults, its sensitivity is low in children. This is why one of tuberculosis research's strategic objectives deals with improving diagnosis [1, 3].

Appropriate diagnosis of active TB is the basis for a timely treatment and control of the disease, which could drastically reduce the mortality associated with paediatric TB [3]. The classical bacteriological assays (culture medium method, ZN staining, and RX findings) showed low sensitivity (Table 2), mainly in indigenous children. Additionally, these techniques have limitations, i.e., scarcity of trained personnel that can interpret chest X-ray or the smear correctly; conventional culture methods can take up to eight weeks to obtain an isolate and determine its susceptibility to antibiotics [23, 24].

The variable clinic presentation and the nonspecific nature of most symptoms complicate the diagnosis of pulmonary TB in children; additionally, several common diseases during childhood can mimic TB clinical presentation; symptoms such as cough, fever, and poor weight gain are common in young children (in particular in poor socioeconomic settings where TB is often endemic) and caused by other conditions such as nonpulmonary TB or nonpulmonary diseases [25].

Concerning synthetic peptides evaluated in this study, a satisfactory diagnostic performance in terms of sensitivity and specificity was found for Ag85A P-29879 in indigenous children, as well as ESAT-6 P-12036 and P-12037 in Creole children, thus showing the potential value of these three peptides as a tool for early active TB diagnosis (Table 3). Indirect ELISA based on synthetic peptides has great advantages due to its low-price, easy standardisation, and implementation for TB detection use, mainly in regions where access to diagnostic tools is limited [26].

High levels of Ag85 complex proteins have been reported in patients with active TB. Their detection in immunoassays has proven to be highly specific, reason why their use in TB screening tests could actually have advantages as the easy implementation, fast diagnosis, and low price [27, 28]. Peptides derived from Ag85 protein have thus been used as a diagnostic tool [26]; here, the Ag85A P-29879 peptide displayed statistically significant high IgG reactivity and a high performance regarding operative characteristics (Figure 1 and Table 3). However, it was not possible to establish a clear cut off value for this peptide that could allow classifying children with TB from healthy ones. Adjustments to the protocol should thus be made, to use this peptide as a diagnostic tool in the future.

ESAT-6 is a well-studied antigen which has proven to be highly antigenic and is recognised by a high percentage of the TB-ill population. This behaviour has been observed in both humans and animals susceptible to mycobacterial infections [29–31]. Previous studies in Creole adults have shown that ESAT-6 peptides P-12033 and P-12034 can improve their sensitivity and specificity levels, reason why they could be useful for diagnosing pulmonary and extrapulmonary tuberculosis [21]. In this study, the peptides P-12036 y P-12037 displayed satisfactory operative performance for this same protein (Table 3), as well as an adequate classification of creole unhealthy and healthy population (Figure 2). Several studies have demonstrated that derived peptides from ESAT-6 displayed a satisfactory sensitivity for pulmonary TB infection diagnosis [32, 33] which agrees with our results and highlighted new possibilities for TB diagnosis and screening in Creole children.

Antigen recognition is a key point regarding the use of antibodies and the development of a serological test; results showed that according to the evaluated group (indigenous or creole), peptide's diagnostic behaviour varied (Table 3, Figures 1 and 2). Immunological studies about Warao population have confirmed Dw inheritance and segregation, i.e., a class II human leukocyte antigen (HLA) including Dw 8.3, DW 16, and Dw22, defined only by homozygous typing of Warao-derived cells [34]. HLA class I and class II allele and haplotype distribution amongst Venezuelan Creoles has shown that an ethnic mixture of Asian, African, and European genes have created a distinctive HLA genetic profile in such hybrid Creole population [35], which could explain the performance of synthetic peptides according to the population group.

Many attempts have been made to develop a serological TB test; however, a simple, inexpensive, point-of-care test is still not available. Such test must discriminate active TB from healthy individuals (noninfected + latent TB), avoid cross-reactivity with the Bacillus Calmette-Guérin (BCG) vaccine or nontuberculous mycobacteria and perform consistently in genetically and immunologically diverse populations [27, 28].

In the present work, IgG reactivity differences against peptides were observed between Warao and Creole child patients. In Creole populations, the results showed a high sensitivity and IgG reactivity against ESAT-6 P-12036: 61 to 80 aa and P-12037: 76 to 95 aa peptides, whilst the Warao child patients showed a high sensitivity and IgG reactivity against Ag85A P-29879: 23 to 43 aa and P-29880: 204 to 223 aa peptides (Table 3). A potential limitation of the present study was that controls did not include children having other pulmonary or nonpulmonary diseases different to TB. A BLAST analysis screening for matches in other pathogens was thus carried out to predict specificity of those peptides showing the best operative characteristics; for those matches with 100% identity, 76-82% corresponded to *Mycobacterium* species (mainly *M. tuberculosis*) for ESAT-6 and 93-100% corresponded to *M. tuberculosis* for Ag85A (data not shown).

This study compared active TB cases versus healthy cases (this group included noninfected + latent TB), previous studies that compare such groups have been reported [11, 36]. However, the present study lacks non-TB disease controls and other pulmonary or nonpulmonary ill individuals, and therefore, the study could not evaluate the clinical performance of this serological test in different population groups. Additional studies that include a broader control group (as above mentioned) may be a good strategy to improve the performance of the serological test and provide further support to the conclusions drawn from this study.

In spite of the promising results, further studies are required to establish if serological differences could be related to intrinsic biological characteristics to the ethnic groups here analysed. In addition, it had been reported that Mtb antigen combinations or an optimal multiantigen cocktail should thus be designed to cover antibodies response heterogeneity and thus ensure the highest possible test sensitivity [37, 38]. Accurate and timely diagnosis of TB remains challenging; most studies have been conducted in adults, so that diagnostic for paediatric TB remains a goal to achieve.

This study's findings confirmed humoral immune response variability regarding Mtb which must be considered when developing serological tests to optimise sensitivity, especially regarding populations having different genetic backgrounds. Indigenous people have usually been reported as having a higher prevalence of several TB determinants than nonindigenous people [39]. Interest has been shown in developing antibody-based diagnostic procedures using multiple antigens to achieve high sensitivity/specificity levels [40, 41]; the morbidity and mortality associated with paediatric TB could thus become drastically reduced if case detection were to be improved and preventative therapy and curative treatment made more rapidly accessible.

## 5. Conclusions

In conclusion, detecting specific-IgG against ESAT-6 and Ag85A peptides in patients' sera gave a reliable active TB diagnosis; this approach holds promise; however, it is worth noting that these results are preliminary, since further inclusion and analysis of samples from patients having other pathologies and coinfections with nontuberculous mycobacteria (i.e., *M. kansasii*, *M. marinum*, *M. ulcerans*, and *M. bovis*) that confirm the conclusions here obtained will allow establishing whether these peptides can be used for diagnosing active pulmonary and extrapulmonary TB, thus contributing to the development of a complementary tool for rapid TB diagnosis in children.

## Figures and Tables

**Table 1:** ESAT-6 and Ag85A protein sequences. Amino acid sequences for synthetic, nonoverlapping peptides spanning ESAT-6 and Ag85A protein sequences. Peptide names and aa numbers are listed on the left-hand side.

	Peptide	aa	Sequence
ESAT-6	P1 (12033)	(1-20)	MTEQQWNFAGIEAAASAIQG
	P2 (12034)	(21-40)	NVTSIHSLLDEGKQSLTKLA
	P3 (12035)	(41-60)	AAWGGSGSEAYQGVQQKWDA
	P4 (12036)	(61-80)	TATELNNALQNLARTISEAG
	P5 (12037)	(76-95)	ISEAGQAMASTEGNVTGMFA

Ag85A	29878	(1-22)	MQLVDRVRGAVTGMSRRLVVGAY
	29879	(23-43)	VGAALVSGLVGAVGGTATAGAY
	10993	(44-63)	FSRPGLPVEYLQVPSPSMGR
	10994	(64-83)	DIKVQFQSGGANSPALYLLD
	10995	(84-104)	GLRAQDDFSGWDINTPAFEWY
	10996	(104-123)	YDQSGLSVVMPVGGQSSFYS
	10997	(124-143)	DWYQPACRKAGCQTYKWETF
	10998	(144-163)	LTSELPGWLQANRHVKPTGSY
	10999	(164-182)	AVVGLSMAASSALTLAIYH
	11000	(183-203)	PQQFVYAGAMSGLLDPSQAMG
	29880	(204-223)	PTLIGLAMGDAGGYKASDMW
	11002	(224-243)	GPKEDPAWQRNDPLLNVGKLY
	11003	(244-263)	IANNTRVWVYCGNGKPSDLG
	11004	(264-283)	GNNLPAKFLEGFVRTSNIKFY
	11005	(284-303)	QDAYNAGGRHNGVFDFPDSG
	11006	(304-322)	THSWEYWGAQLNAMKPDLQ
	29881	(320-338)	DLQRALGATPNTGPAPQGAY

**Table 2:** The children's demographic characteristics. Child patients having pulmonary TB: Warao children (WPCh) and Creole children (CPCh). Healthy control children with no suspicion of tuberculosis: Warao indigenous (WCCh) and Creole children (CCCh).

Characteristic	Patients		Controls	
	WPCh	CPCh	WCCh	CCCh
Age	7.5 ± 4.2	5.2 ± 3.6	8.6 ± 2.3	6.6 ± 2.4
Female (%)	41.0	46.1	60.5^(a)^	39.0^(b)^
Male (%)	58.9	53.8	39.4^(c)^	60.9^(d)^
TST+ (%)	74.0^(e)^	45.5^(f)^	20.1^(g)^	ND
Smear + (%)	3.7	0	0	0
Culture+ (%)	7.4	0	0	0
Chest X-ray (%)	59.3^(h)^	100.0^(i)^	0	0

There was a statistically significant difference between females and males (a) and (b) (*p* < 0.0001) and (c) and (d) (*p* < 0.02). There was a statistically significant difference between (e) and (f) and (g) (*p* < 0.0001) concerning TST+ status and a statistically significant difference between (h) and (i) (*p* < 0.007) regarding findings related to radiographic characteristics concerning lesions suggestive of active TB. ND: test was not performed.

**Table 3:** Antipeptide test diagnostic accuracy.

	Peptide	Sensitivity (%)	Confidence interval	Specificity (%)	Confidence interval	Positive likelihood ratio	Negative likelihood ratio	Positive predictive value	Negative predictive value
Indigenous	**29880**	100	90.4-100.0	—	—	1.26	0.00	37.8	100
	**29879**	94.6	81.8-99.2	—	—	1.87	0.11	47.3	95.0
	**10997**	94.6	81.8-99.2	—	—	1.14	0.32	35.4	86.7
	**11003**	—	—	97.4	90.9-99.6	12.5	0.69	85.7	75.0
	**11004**	—	—	93.5	85.5-97.8	7.91	0.52	79.2	80.0

Creole	**12036**	100	75.1-100.0	—	—	3.94	0.00	44.8	100
	**12037**	92.3	63.9-98.7	—	—	2.08	0.14	30.0	97.2
	**12035**	84.6	54.5-97.6	—	—	2.42	0.24	33.3	95.3
	**12033**	—	—	95.3	86.7-99.0	8.08	0.65	62.5	88.2
	**12034**	—	—	93.8	84.5-98.2	7.27	0.57	60.0	89.4

## Data Availability

The data used to support the findings of this study are available from the corresponding author on reasonable request.

## Abbreviations

TB: Tuberculosis
ROC: Receiver-operating characteristic
AUC: Area under curve
CI: Confidence interval
WHO: World Health Organization
MDR-TB: Multidrug-resistant tuberculosis
Mtb: Mycobacterium tuberculosis
TST: Tuberculin skin test
ELISA: Enzyme-linked immunosorbent assay
IgG: Immunoglobulin G
OD: Optical density.

## Supplementary Materials

Supplementary Materials Figure S1: chest X-ray studies of children suspected of suffering tuberculosis. (a) A Warao indigenous child's X-ray. Widening of the mediastinum, suggesting parahilar metastatic adenopathy can be observed on the right-hand side (arrowheads) and pulmonary parenchymal involvement in the form of right-hand side bilateral interstitial pattern predominance. There is no pleural effusion. (b) A Creole child's thorax X-rays. Mediastinal widening can be observed, suggestive of hilar adenopathy (arrowhead), and parenchymal involvement tends to form a consolidating alveolar pattern in the right pulmonary base (asterisk). Figure S2: anti-Ag85 and anti-ESAT-6 peptide tests' ROC curves. ROC analysis for evaluating Ag85A-derived synthetic peptides (P-29879 and P-11003) for distinguishing indigenous patients from indigenous controls. ROC analysis for evaluating ESAT-6-derived synthetic peptides (P-12036 and P-12033) for distinguishing Creole patients from Creole controls. AUC: area under the curve; SE: standard error; 95% CI: 95% confidence interval.
