# Supplementary figures and images for: ESAT-6 and Ag85A Synthetic Peptides as Candidates for an Immunodiagnostic Test in Children with a Clinical Suspicion of Tuberculosis
Source: Dis Markers. 2021 Jul 6;2021:6673250. doi: 10.1155/2021/6673250 (PMC8279849; doi:10.1155/2021/6673250)

(a)

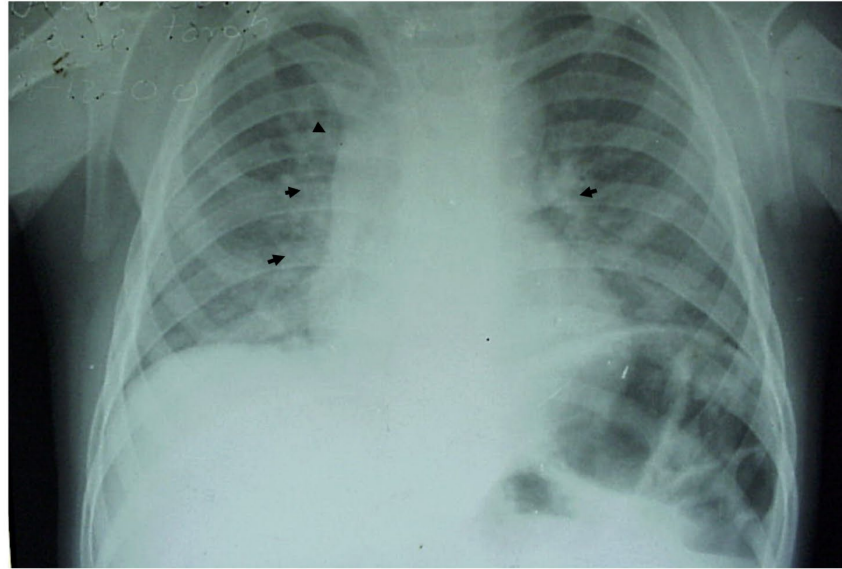

(b)

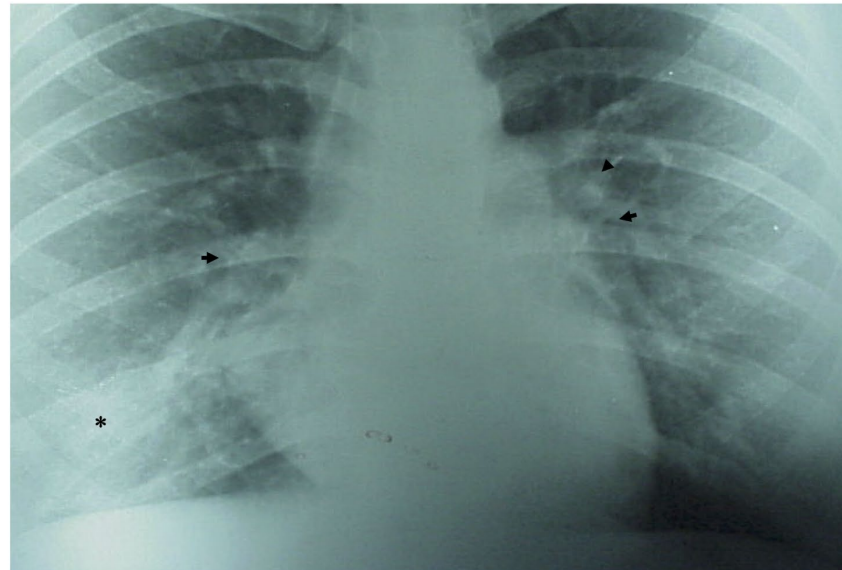

**Figure S1**

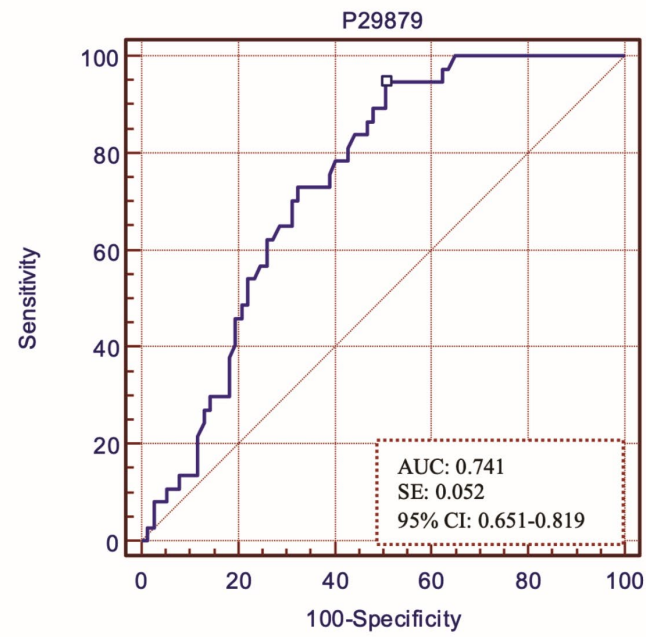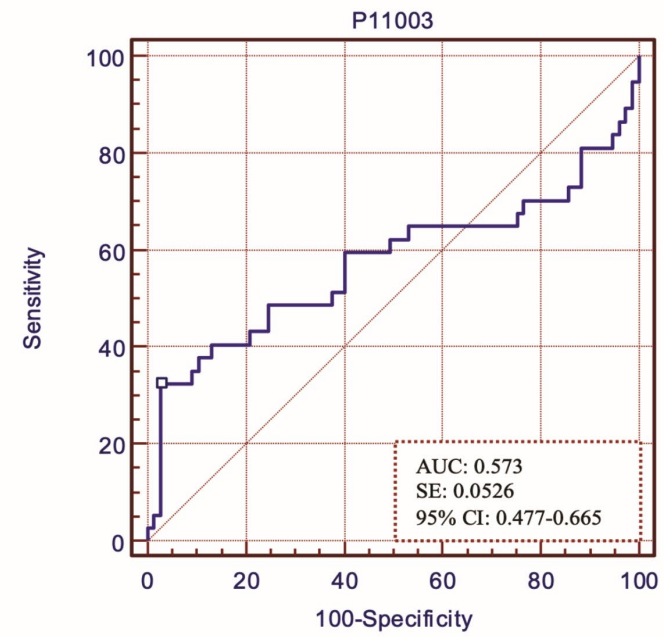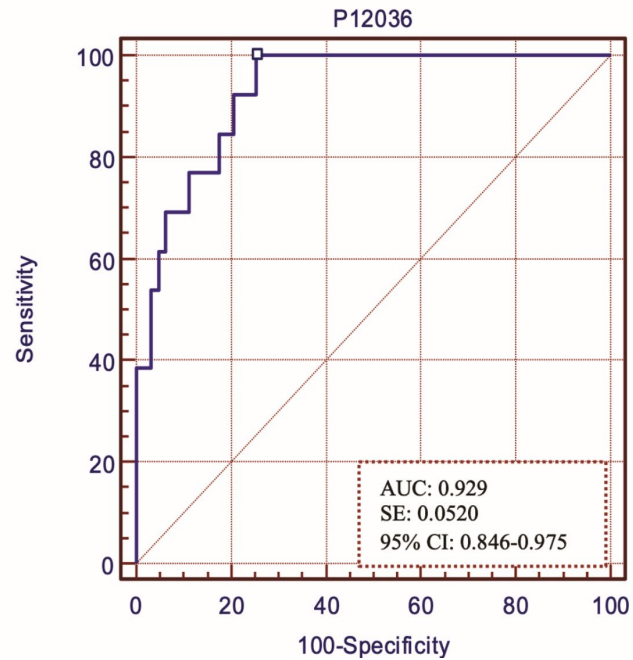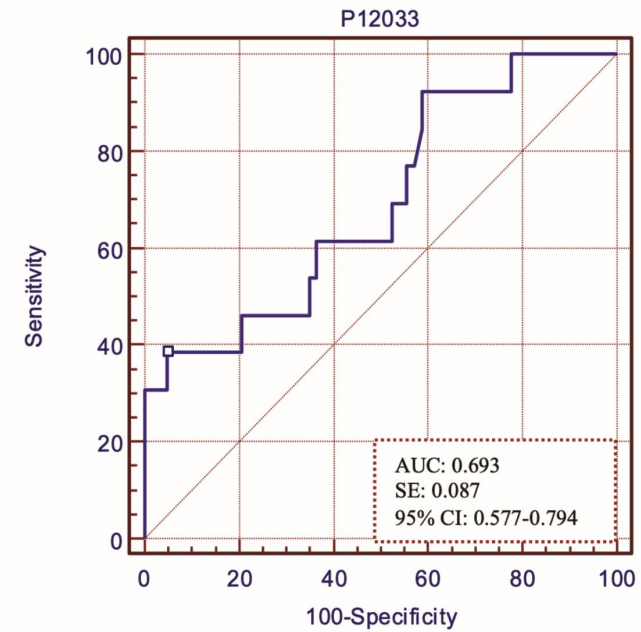

**Figure S2**

Supplement: Supplementary Materials — Figure S1: chest X-ray studies of children suspected of suffering tuberculosis. (a) A Warao indigenous child's X-ray. Widening of the mediastinum, suggesting parahilar metastatic adenopathy can be observed on the right-hand side (arrowheads) and pulmonary parenchymal involvement in the form of right-hand side bilateral interstitial pattern predominance. There is no pleural effusion. (b) A Creole child's thorax X-rays. Mediastinal widening can be observed, suggestive of hilar adenopathy (arrowhead), and parenchymal involvement tends to form a consolidating alveolar pattern in the right pulmonary base (asterisk). Figure S2: anti-Ag85 and anti-ESAT-6 peptide tests' ROC curves. ROC analysis for evaluating Ag85A-derived synthetic peptides (P-29879 and P-11003) for distinguishing indigenous patients from indigenous controls. ROC analysis for evaluating ESAT-6-derived synthetic peptides (P-12036 and P-12033) for distinguishing Creole patients from Creole controls. AUC: area under the curve; SE: standard error; 95% CI: 95% confidence interval. [file 6673250.f1.pdf]
